# Tropism of engineered and evolved recombinant AAV serotypes in the *rd1* mouse and *ex vivo* primate retina

**DOI:** 10.1038/gt.2017.85

**Published:** 2017-11-16

**Authors:** D G Hickey, T L Edwards, A R Barnard, M S Singh, S R de Silva, M E McClements, J G Flannery, M W Hankins, R E MacLaren

**Affiliations:** 1 Nuffield Laboratory of Ophthalmology, University of Oxford, Oxford, UK; 2 Moorfields Eye Hospital NHS Foundation Trust NIHR Biomedical Research Centre, London, UK; 3 Helen Wills Neuroscience Institute, University of California, Berkeley, Berkeley, CA, USA; 4 Sleep and Circadian Neuroscience Institute, University of Oxford, Oxford, UK; 5 Oxford University Hospitals NHS Trust Biomedical Research Centre, Oxford, UK

## Abstract

There is much debate on the adeno-associated virus (AAV) serotype that best targets specific retinal cell types and the route of surgical delivery—intravitreal or subretinal. This study compared three of the most efficacious AAV vectors known to date in a mouse model of retinal degeneration (*rd1* mouse) and macaque and human retinal explants. Green fluorescent protein (GFP) driven by a ubiquitous promoter was packaged into three AAV capsids: AAV2/8(Y733F), AAV2/2(quad Y-F) and AAV2/2(7m8). Overall, AAV2/2(7m8) transduced the largest area of retina and resulted in the highest level of GFP expression, followed by AAV2/2(quad Y-F) and AAV2/8(Y733F). AAV2/2(7m8) and AAV2/2(quad Y-F) both resulted in similar patterns of transduction whether they were injected intravitreally or subretinally. AAV2/8(Y733F) transduced a significantly smaller area of retina when injected intravitreally compared with subretinally. Retinal ganglion cells, horizontal cells and retinal pigment epithelium expressed relatively high levels of GFP in the mouse retina, whereas amacrine cells expressed low levels of GFP and bipolar cells were infrequently transduced. Cone cells were the most frequently transduced cell type in macaque retina explants, whereas Müller cells were the predominant transduced cell type in human retinal explants. Of the AAV serotypes tested, AAV2/2(7m8) was the most effective at transducing a range of cell types in degenerate mouse retina and macaque and human retinal explants.

## Introduction

Inherited retinal degenerations are a leading cause of blindness in the working-age population of industrialised countries.^1^ Gene therapy is a therapeutic approach that has great potential to slow or reverse blinding retinal degeneration by delivering a normal copy of a mutated gene^2, 3^ (gene supplementation), editing the mutated gene^4^ (for example, using CRISPR/Cas9), knocking down the expression of a mutant allele using RNA interference^5^ or expressing neuroprotective factors.^6^

Adeno-associated virus (AAV) is the vector of choice for most retinal gene therapy applications where the transgene is relatively small because of its established record of safety and efficacy in preclinical studies and clinical trials.^2, 3, 7, 8^ The efficacy of a vector is measured by both the efficiency with which the genetic cargo is delivered and its specificity for the target cell type (its tropism).

A greater understanding of AAV biology has led to the generation of rationally designed recombinant AAV serotypes. Mutation of surface tyrosine (Y) residues to phenylalanine (F) was found to reduce the rate of proteasome-mediated degradation and to significantly increase transgene expression *in vitro* and *in vivo*, allowing a comparable transgene expression level to be achieved with an ∼10-fold lower AAV dose.^9^ Subsequent work showed further increased transduction efficiency and a wider tropism by mutating two to seven surface tyrosine residues of AAV2/2.^(ref. 10)^

AAV2/8, a serotype originally isolated from rhesus monkeys,^11^ is effective at transducing photoreceptors and retinal pigment epithelium more efficiently than AAV2/2 and AAV2/5 following subretinal injection into nondegenerate mouse eyes.^12^ In additionally, AAV2/8 transduced photoreceptors of cynomolgus monkeys more efficiently than AAV2/2 following subretinal injection.^13^ Building on the findings from AAV2/2 site-directed tyrosine to phenylalanine mutations, a capsid-mutant AAV2/8 serotype termed AAV2/8(Y733F) was developed that transduced more cells and demonstrated significantly higher transgene expression compared with wild-type AAV2/8.^(ref. 14)^ Subretinal injections of AAV2/8(Y733F) into nondegenerate mouse eyes led to stronger and more widespread green fluorescent protein (GFP) signal, compared with wild-type and mutant AAV2/2, AAV2/8 and AAV2/9 variants.

In contrast to the targeted mutation approach, an *in vivo* directed evolution strategy was used in mice to create novel AAV serotypes that are more efficient at transducing murine photoreceptors after being injected intravitreally.^15^ AAV2/2(7m8), an evolved variant that features a 7-amino-acid sequence inserted after position 587 of capsid protein VP1,^15, 16^ was selected for *in vivo* studies in mice and macaque and showed strong expression across the retina and in all major classes of retinal cells.^15^

The three serotypes selected for this study were: AAV2/8(Y733F), AAV2/2(Y272, 444, 500, 730F) (abbreviated to ‘quad Y-F’) and AAV2/2(7m8). Data directly comparing these leading AAV vectors in degenerate retina are lacking. AAV2/8(Y733F) was selected as a previous comparative study showed this to have the greatest transgene expression intensity and transduction area following subretinal delivery to nondegenerate mouse retina compared with other AAV2/2, AAV2/8 and AAV2/9 serotypes.^14^ AAV2/8(Y733F) in conjunction with a ubiquitous promoter has also been demonstrated to transduce bipolar cells in the *rd1* mouse.^17^ Of the AAV2/2 capsid mutants, AAV2/2(quad Y-F) was chosen for further assessment as, when tested in nondegenerate mouse retina, it has been found to transduce photoreceptors following intravitreal injection, to occasionally transduce retinal bipolar cells when delivered into the subretinal space and, overall, to demonstrate a combination of high levels of transgene expression and a diversity of transduced cell types that was not matched by other AAV2/2 capsid mutants.^10^ Finally, AAV2/2(7m8) was selected as it represents a contrasting approach to AAV development, has demonstrated potent transduction across the retina in nondegenerate mice and macaque retinas^15^ and has also been shown to effectively transduce bipolar cells—a particularly challenging cell target to transduce.^18^

This research aimed to test these three AAV serotypes in a mouse model of retinal degeneration together with macaque and human explants *in vitro* to inform AAV serotype selection for basic and translational retinal research. As the end-stage degenerate retina is the target tissue for a number of gene therapy strategies, including optogenetic vision restoration,^19^ we compared the transduction profile of the three recombinant AAV serotypes in a model of retinal degeneration, the *rd1* mouse, that has a naturally occurring nonsense mutation of the rod-specific *phosphodiesterase 6B (Pde6b)* gene.^20, 21^ In addition to the loss of photoreceptors, the degenerate retina undergoes many structural, physiological and gene expression changes that makes it distinct from the nondegenerate retina^22, 23, 24, 25^ and can cause changes in AAV spread and transduction efficiency.^26^ This *in vivo* model enabled the comparison of the area, intensity and cells types transduced by the three AAV vectors following both intravitreal and subretinal injections. Clinical data directly comparing intravitreal and subretinal injections are lacking. We additionally tested the three AAV serotypes in macaque and human explants to see whether species-specific tropism differences that have been demonstrated by *in vivo* studies^27^ were evident. The use of retinal tissue from healthy macaque retina and degenerate human retina provided some insight into disease-specific tropism differences. Comparison with *in vivo* studies enabled an assessment of the utility of retinal explants as models of *in vivo* transduction.

This study found that intravitreal and subretinal injections were similarly effective for AAV2/2(quad Y-F) and AAV2/2(7m8), but the subretinal route was more effective for AAV2/8(Y733F). All major retinal cell types of the *rd1* mouse retina were transduced, with ganglion cells, horizontal cells and retinal pigment epithelium well transduced, whereas bipolar cells were sparsely transduced. In macaque and human retinal explants, AAV2/2(7m8) transduced the greatest number of cells. These data support the use of AAV2/2(7m8) in mouse models as well as primate retina.

## Results

### AAV tropism in *rd1* mice, a model of retinal degeneration

#### Intravitreal AAV2/2(7m8) transduces the greatest area of mouse retina

Three recombinant AAV vectors were produced by packaging the same expression cassette, consisting of GFP driven by a ubiquitous CAG synthetic promoter with an SV40 poly(A) sequence, into three different AAV capsids: AAV2/8(Y733F), AAV2/2(quad Y-F) and AAV2/2(7m8). At 3 weeks after injection with one of the three AAV-GFP test vectors, *rd1* mice underwent *in vivo* confocal scanning laser ophthalmoscopy to quantify the distribution and intensity of GFP expression in the retina.

**Figure 1:**
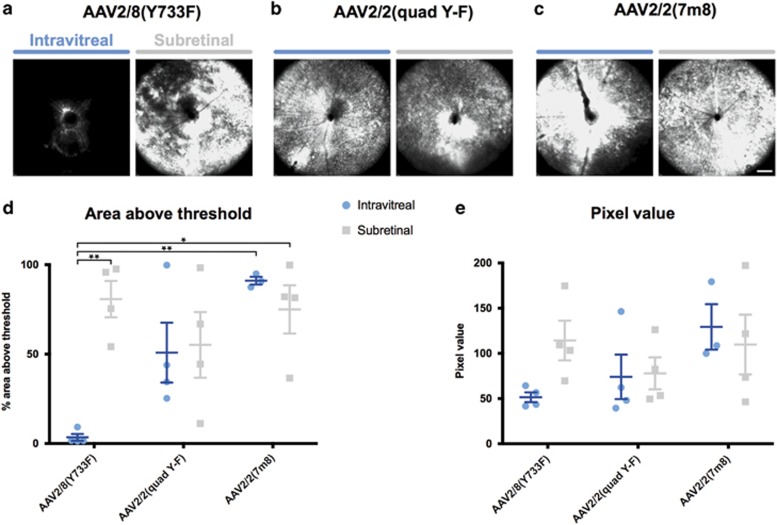
Scanning laser ophthalmoscopy of degenerate mouse retinas injected with three GFP-expressing AAV serotypes. (**a**–**c**) Mice were injected at 13–14 weeks of age and assessed 3 weeks post injection by confocal scanning laser ophthalmoscopy (cSLO). Images centred on the optic disc were acquired from each of the eyes injected with each of the following serotypes: (**a**) AAV2/8(Y733F), (**b**) AAV2/2(quad Y-F) and (**c**) AAV2/2(7m8). The same autofluorescence settings were used. (**d**) SLO images were quantified by setting a threshold pixel value and determining the area of each image above this threshold and (**e**) the intensity of the signal (‘Pixel value’) in the area above the threshold (mean±s.e.m.; *n*=4, AAV2/8(Y733F) and AAV2/2(quad Y-F); *n*=3, AAV2/2(7m8)). *P<0.05, **P<0.01. Scale bar, 1 mm.

In all three intravitreally injected AAV vector groups the area around the optic disc showed the most fluorescence (Figures 1a–c), in keeping with the high concentration of ganglion cell axons in this region. In eyes injected with AAV2/2(quad Y-F) or AAV2/2(7m8) the fluorescence extended beyond the central 55° to the peripheral retina where it was strongest adjacent to blood vessels. Fluorescence was mostly speckled, with small, highly fluorescent foci.

GFP fluorescence did not appear to be limited to the boundary of the bleb that was created to deliver AAV vector to the subretinal space between the neuroretina and the retinal pigment epithelium. In most eyes the subretinal bleb was limited to two quadrants, but fluorescence was seen at all angles around the optic disc (Figures 1a–c). Despite the AAV vector being delivered by a transchoroidal approach to the subretinal space in this group of eyes, lines of fluorescence from the periphery to the optic disc were notable in some eyes, consistent with GFP expression in ganglion cell axons, indicating that AAV had traversed the retina and transduced retinal ganglion cells in the innermost retinal layer (Supplementary Figure 1).

GFP expression was quantified by assessing the area of GFP expression above the background signal threshold and intensity was compared using mean pixel values (Figures 1d and e). Examining the effect of delivery route (intravitreal or subretinal) and AAV serotype on transduced area showed a statistically significant interaction between AAV serotype and area, F(2, 17)=5.1, *P*=0.019 (Figure 1d; *n*=4, all groups except intravitreal AAV2/2(7m8) (*n*=3)). Simple effects analysis with Bonferroni correction showed that the area of GFP expression from an intravitreal injection with AAV2/8(Y733F) (3.4±1.9%) was significantly lower than each of subretinal AAV2/8(Y733F) (80.8±10.2% *P*<0.01), intravitreal AAV2/2(7m8) (91.1±2.2% *P*<0.01) and subretinal AAV2/2(7m8) (75.0±13.5% *P*<0.05). An ordinary two-way analysis of variance examining the effect of delivery route and AAV serotype on pixel value showed no statistically significant interactions (Figure 1e).

Hence, intravitreal injection with AAV2/8(Y733F) led to the smallest area of transduced retina, but statistically significant differences between other vector/delivery routes were not detected.

#### Injection route determines relative transduction of retinal layers

**Figure 2:**
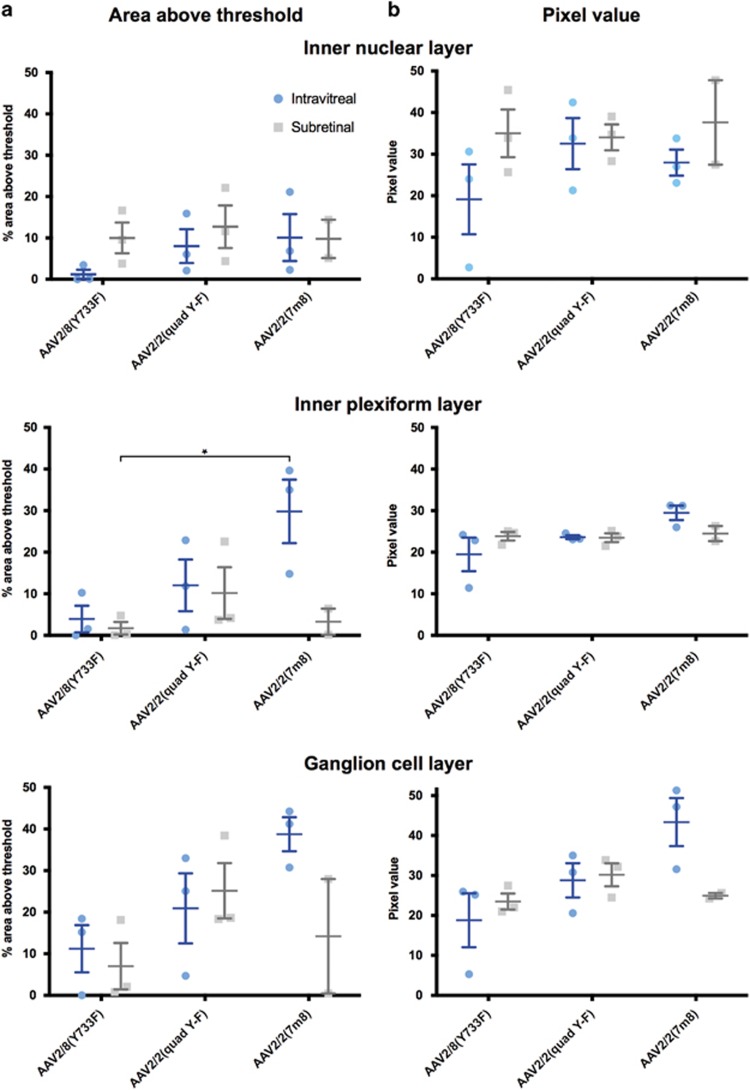
Statistical analysis of immunohistochemistry data comparing delivery routes and AAV serotypes in degenerate *rd1* mouse retinas. (**a**) Ordinary two-way analysis of variance (ANOVA) tests examining the effect of delivery route (intravitreal or subretinal) and AAV serotype on the area above threshold for each of the three layers of the retina were examined. Only the effect of delivery route and AAV serotype on the area above threshold in the inner plexiform layer showed a statistically significant interaction between delivery route and area above threshold, F(1, 11)=5.2, *P*=0.043 (*n*=3, all groups except intravitreal AAV2/2(7 m8) (*n*=2)). Simple effects analysis showed that the area above threshold in the inner plexiform layer from a subretinal injection with AAV2/8(Y733F) was significantly lower than the area associated with an intravitreal injection with AAV2/2(7m8). (**b**) Ordinary two-way ANOVA tests examining the effect of delivery route and AAV serotype on the pixel value in each of the three layers of the retina showed no statistically significant interactions. *P<0.05.

To compare the level of GFP expression across retinal layers, vertical sections of the *rd1* mouse eyes that had received injections of three serotypes of AAV-GFP were prepared. To quantify penetration and transgene expression efficiency the retina was divided into the three remaining layers (inner nuclear layer, inner plexiform layer and ganglion cell layer) and the percentage area above the background signal and the mean pixel value within the area above threshold was calculated (Figure 2).

Of those eyes that received an intravitreal injection, AAV2/2(7m8) produced the greatest area above threshold in all three layers of the degenerate retina, with 10.1±5.7, 29.8±7.6 and 38.7±4.1% of the inner nuclear layer, inner plexiform layer and ganglion cell layer, respectively, above the threshold (Figure 2a). The area transduced by AAV2/2(7m8) in the inner plexiform layer and ganglion cell layer was notably higher than the transduced areas from AAV2/8(Y733F) (3.9±3.2 and 11.2±5.7%, respectively) and AAV2/2(quad Y-F) (12.0±6.2 and 20.9±8.4%, respectively) intravitreal injections. In the inner nuclear layer, AAV2/2(quad Y-F) and AAV2/2(7m8) groups had comparable areas transduced with 8.0±4.1 and 10.1±5.7%, respectively. The AAV2/8(Y733F) group in contrast had 1.1±1.1% of its inner nuclear layer above threshold. Consistent with the confocal scanning laser ophthalmoscopy results, an intravitreal injection with AAV2/8(Y733F) was not as effective as the other two serotypes, with the least area transduced in all layers of the degenerate retina.

A different pattern of GFP expression in the retinal layers was evident following subretinal delivery of AAV vector. Generally, subretinal injections resulted in more of the inner nuclear layer and a smaller area of the ganglion cell and inner plexiform layers being above threshold compared with retinas that had received the same AAV by the intravitreal route (Figure 2a). A notable exception to this pattern was with the use of AAV2/2(quad Y-F) where the area of the ganglion cell layer above threshold was higher following subretinal (25.1±6.6%) versus intravitreal (20.9±8.4%) vector delivery (Figure 2a).

There was a statistically significant interaction between delivery route and area above threshold in the inner plexiform layer, F(1, 11)=5.2, *P*=0.043 (*n*=3, all groups except intravitreal AAV2/2(7m8) (*n*=2); Figure 2a). Simple effects analysis showed that the area in the inner plexiform layer from a subretinal injection with AAV2/8(Y733F) (1.7±1.5%) was significantly (*P*<0.05, Bonferroni correction) lower than the area above threshold because of an intravitreal injection with AAV2/2(7m8) (29.8±7.6%). Ordinary two-way analysis of variance tests examining the effect of delivery route and AAV serotype on the pixel value in each of the three layers of the retina showed no statistically significant interactions (Figure 2b).

#### Horizontal cells, ganglion cells and retinal pigment epithelium are strongly transduced by all three serotypes

**Figure 3:**
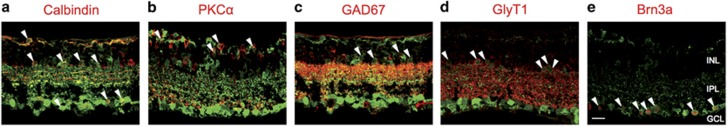
Confocal fluorescence micrographs of degenerate mouse retinas injected with three serotypes of AAV-GFP and double labelled for GFP and retinal cell markers. Degenerate *rd1* mouse retinas were injected either intravitreally or subretinally with an AAV vector expressing GFP driven by a ubiquitous promoter, with one of three AAV serotypes: AAV2/8(Y733F), AAV2/2(quad Y-F) or AAV2/2(7m8). Vertical sections were double immunolabelled for GFP and retinal cell markers: (**a**) calbindin; (**b**) protein kinase-Cα (PKCα); (**c**) glutamate decarboxylase 67 (GAD67); (**d**) glycine transporter 1 (GlyT1) or (**e**) brain-specific homeobox/POU domain protein 3a (Brn3a). Cell bodies that were immunopositive for both GFP and the cell marker are encircled. Arrowheads indicate GFP-expressing retinal pigment epithelium (RPE). The RPE cannot be identified in the majority of the panels because of it detaching during sample preparation. See Table 1 for a summary of the colocalisation results. GCL, ganglion cell layer; INL, inner nuclear layer. Scale bar, 20 μm.

To determine which cell types of *rd1* degenerate mouse retinas were transduced by intravitreal or subretinal injection of three serotypes of AAV-GFP, vertical sections were co-labelled with a range of antibody retinal cell markers (Figure 3). The relative transduction efficiency of the different serotypes did not differ greatly — the same cell types were transduced in all cases (Table 1). The route of delivery also did not influence which cell types were transduced; however, there were differences in the level of expression of GFP in the transduced cells depending on the delivery route.

The cell type most strongly transduced in the inner nuclear layer following intravitreal or subretinal AAV vector delivery was the horizontal cell, as identified by their position in the outer part of the inner nuclear layer and positive staining with an anti-calbindin antibody (Figure 3a). In retinas injected intravitreally or subretinally, transduction of rod bipolar cells, identified by protein kinase-Cα (PKCα) immunolabelling, was sparse (Figure 3b). When transduced PKCα-positive cells were identified, their level of GFP expression was very low compared with horizontal cells. A population of amacrine cells were identified by immunolabelling with antibodies against calbindin, glutamate decarboxylase 67 (GAD67) and glycine transporter 1 (GlyT1) (Figures 3a, c and d). Retinas from each of the viral serotypes and delivery routes had amacrine cell marker-positive GFP-expressing cells in the inner half of the inner nuclear layer. Generally, cells that were immunolabelled with an amacrine cell marker were weakly GFP positive as compared with the GFP levels expressed in horizontal and ganglion cells.

Transduced cells in the ganglion cell layer included photosensitive retinal ganglion cells, Brn3a-positive retinal ganglion cells and amacrine cells. The majority of the GFP-positive cells in the ganglion cell layer were Brn3a-positive retinal ganglion cells that generally had a high level of GFP expression compared with other cell types (Figure 3e). Brn3a-positive retinal ganglion cells were observed irrespective of whether the AAV-GFP was injected subretinally or intravitreally. A small number of GFP-positive cells that co-labelled for calbindin or GAD67 were present in the ganglion cell layer, suggesting that some displaced amacrine cells were transduced (Figure 3c).

Sections from a retina that had received an intravitreal injection of AAV2/2(7m8)-GFP were immunolabelled with an anti-mouse melanopsin antibody, demonstrating that AAV2/2(7m8) transduced melanopsin-positive photosensitive retinal ganglion cells (Supplementary Figure 2).

All AAV-GFP vectors studied, regardless of serotype and delivery route, showed high levels of GFP expression in retinal pigment epithelial cells (data not shown).

### AAV tropism in nondegenerate macaque retina

**Figure 4:**
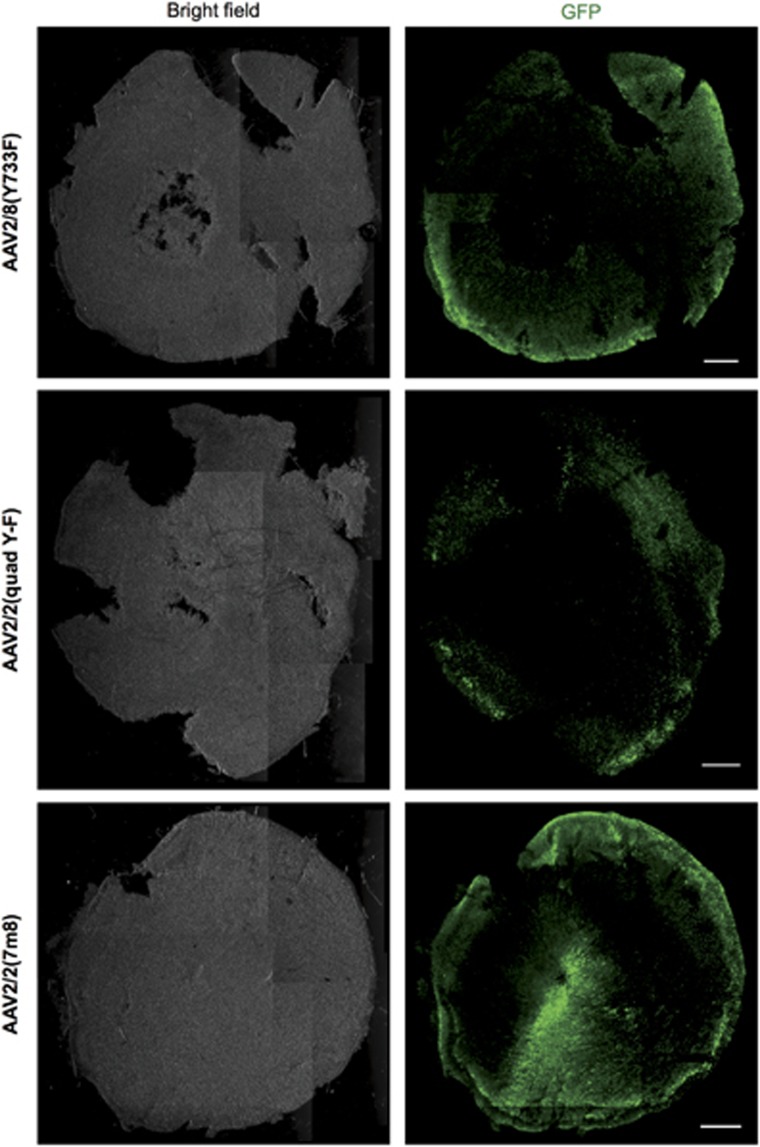
Macaque fovea explants transduced with three serotypes of AAV-GFP. The 3 mm diameter discs of macaque retina centred on the fovea were cultured for 8 days in AAV-GFP solution before being fixed and immunolabelled with anti-GFP antibody. Three different AAV serotypes were used to transduce different explants: AAV2/8(Y733F), AAV2/2(quad Y-F) and AAV2/2(7m8). Images were acquired using the same settings. The outer nuclear layer is nearest the camera in all images. Scale bar, 500 μm.

To compare the transduction pattern in mice to that of a primate, the three serotypes of AAV-GFP were applied to explants of foveal and peripheral retina from rhesus macaques (*Macaca mullata*) *ex vivo*. At 8 days after adding 10^10^ vector genomes (vg) to each explant, GFP expression was evident in immunolabelled explants. GFP expression was strongest in the periphery of the foveal explants, adjacent the cut edge (Figure 4). The AAV2/2(7m8) transduced foveal explant featured higher levels of GFP expression that extended more centrally towards the foveola.

**Figure 5:**
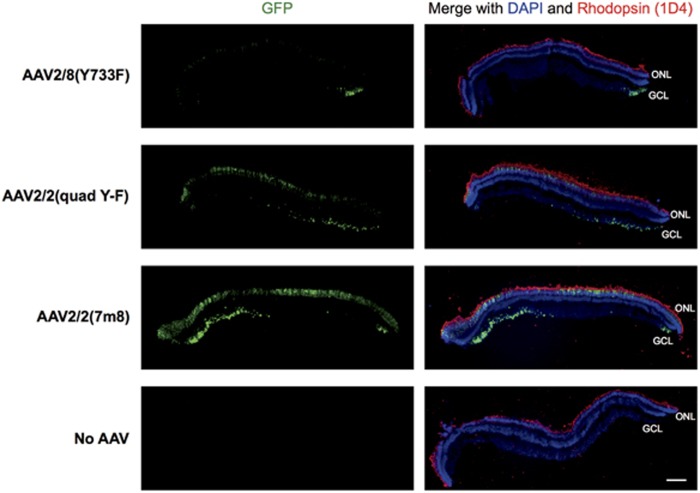
Fluorescence micrographs of foveal macaque retina explants transduced with three serotypes of AAV-GFP and double labelled for GFP and rhodopsin. Macaque explants from the fovea were transduced with AAV-GFP with one of three serotypes—AAV2/8(Y733F), AAV2/2(quad Y-F) or AAV2/2(7m8)—or no AAV. Explants were fixed, sectioned and double immunolabelled with antibodies against GFP and rhodopsin (1D4) to show the distribution of GFP in the retinal layers. All sections are oriented with photoreceptors up. Note that these images are from tissue that is unique from those samples shown in Figure 4. GCL, ganglion cell layer; INL, inner nuclear layer; ONL, outer nuclear layer. Scale bar, 200 μm.

**Figure 6:**
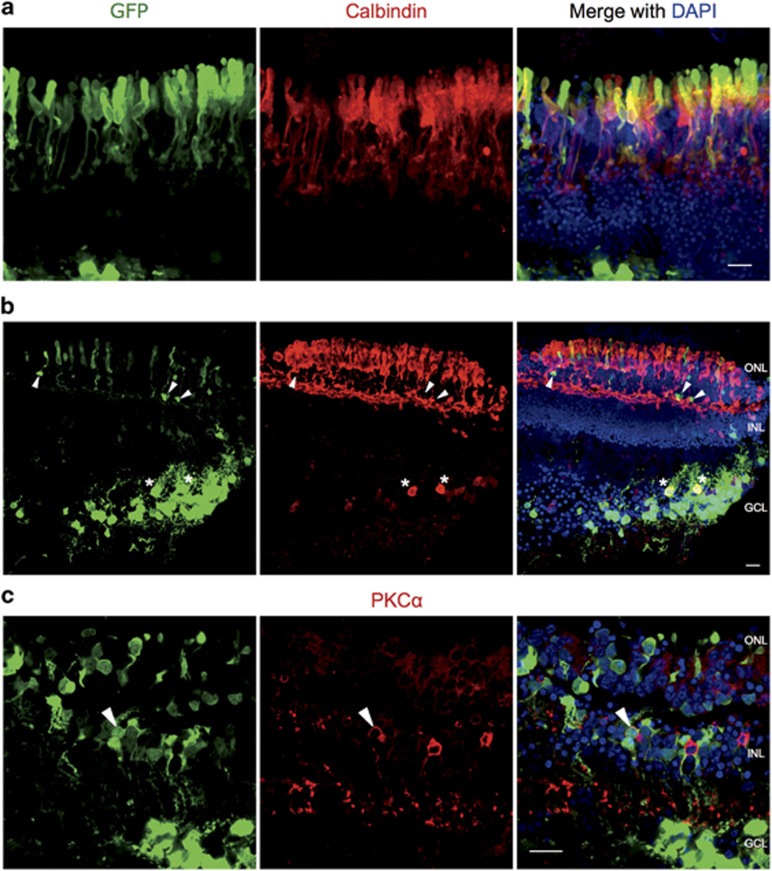
Confocal fluorescence micrographs of macaque foveal explants transduced with AAV2/8(Y733F)-GFP, co-labelled for retinal cell markers. (**a**) The majority of GFP-positive cells in macaque foveal explants were calbindin positive, indicating they were cones. (**b**) However, a small number of GFP-positive photoreceptors were calbindin negative (arrowheads) that, combined with the slender inner segment morphology of these cells and the more inner location of the cell bodies, suggests these are rods. Calbindin- and GFP-positive cells were also identified in the ganglion cell layer (*). (**c**) At the very periphery of a flat-mounted explant, a PKCα-positive bipolar cell was co-labelled (arrowhead). GCL, ganglion cell layer; INL, inner nuclear layer; ONL, outer nuclear layer. Scale bar, 20 μm.

The pattern of cell types transduced was similar across the three AAV serotypes, but AAV2/2(7m8) transduced explants had higher levels of GFP expression spread over a larger area of retina (Table 1). Vertical sections of foveal and peripheral retina explants were immunolabelled for GFP and rhodopsin (anti-1D4 antibody). Foveal explants were found to have significantly more widespread and higher levels of GFP expression than peripheral retina explants transduced with the same AAV serotype (Figure 5; peripheral explant data not shown). The outer nuclear layer contained the highest number of GFP-positive cells for all three serotypes. Some explants had a cluster of GFP-positive cells in the ganglion cell layer, but only towards an edge of the section (Figure 5). Colocalisation studies showed that a large majority of GFP-positive cells were cones—regardless of the serotype of the AAV-GFP vector, calbindin colocalised with almost all of the GFP-positive cells in the outer nuclear layer (Figure 6a). Only a small number of GFP-positive, calbindin-negative cell rods were identified in the inner part of the outer nuclear layer (Figure 6b).

GFP-positive cells in the ganglion cell layer were restricted to the edge of the explants, where tissue damage was caused when the explant disc was cut from the retina. Some of the GFP-positive cells in the ganglion cell layer were noted to be Brn3a positive (data not shown), whereas others were calbindin positive (Figure 6b). In addition, at the periphery of a foveal section, a GFP-positive cell in the inner nuclear layer was found to co-label for PKCα (Figure 6c).

### AAV tropism in human retinal explants

Explants of human retina fragments were also transduced with the three different AAV-GFP serotypes. Human retina was acquired from patients who required retinectomy during retinal detachment surgery (Supplementary Figure 3). Hence, apart from species differences, the human tissue was degenerate, providing additional information relevant to the clinical scenario. At 7 days after immersing the explants in media containing 1.67 × 10^10^ vg per ml of the AAV serotypes, the human retina samples were fixed and immunolabelled for GFP and retinal cell markers.

**Figure 7:**
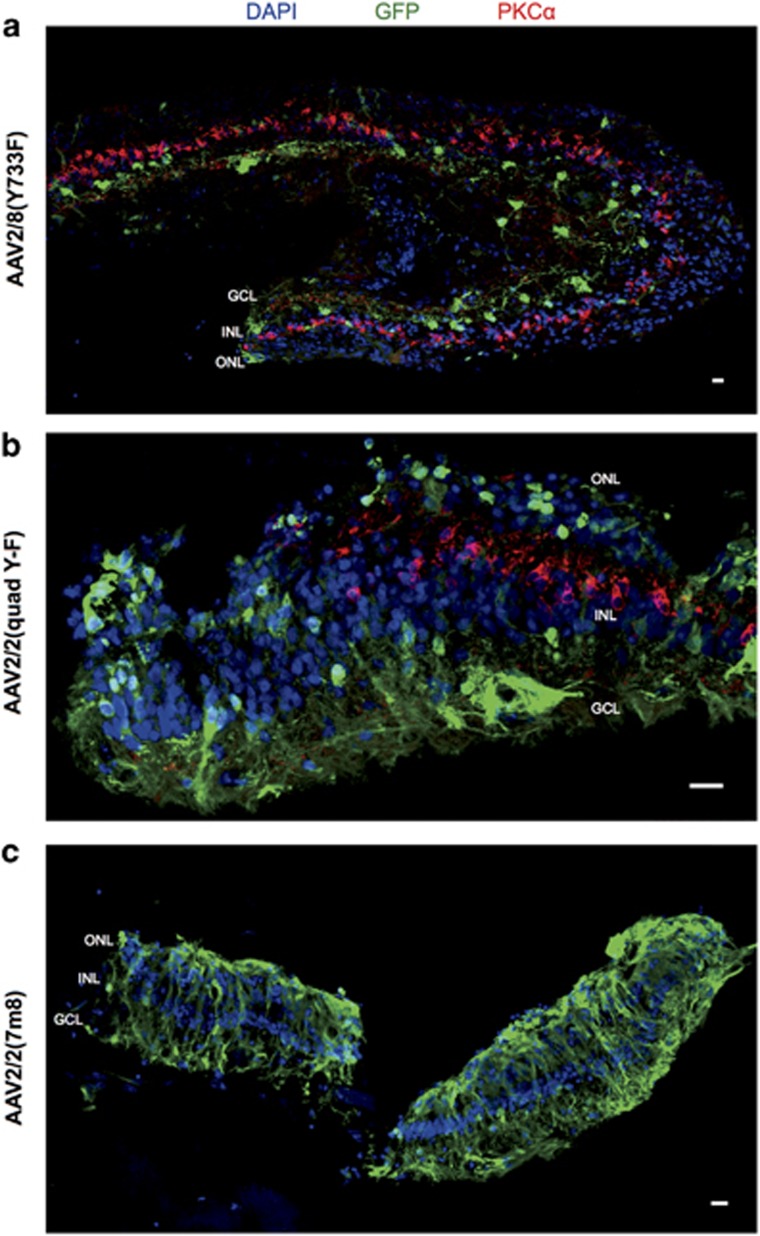
Fluorescence micrographs of human retina explants transduced with three serotypes of AAV-GFP. Retina from patients requiring retinectomy was cultured for 1 week in the presence of AAV-GFP of three different serotypes: (**a**) AAV2/8(Y733F), (**b**) AAV2/2(quad Y-F) or (**c**) AAV2/2(7m8). Explants were sectioned and immunolabelled for GFP and cell markers, such as protein kinase-Cα (PKCα, used in (**a**) and (**b**) only). GFP was largely limited to the inner nuclear layer (INL) of AAV2/8(Y733F) transduced retina, but extended throughout all retinal layers in retina transduced with AAV2/2(quad Y-F) and AAV2/2 (7m8). GCL, ganglion cell layer; ONL, outer nuclear layer. Scale bar, 20 μm.

**Figure 8:**
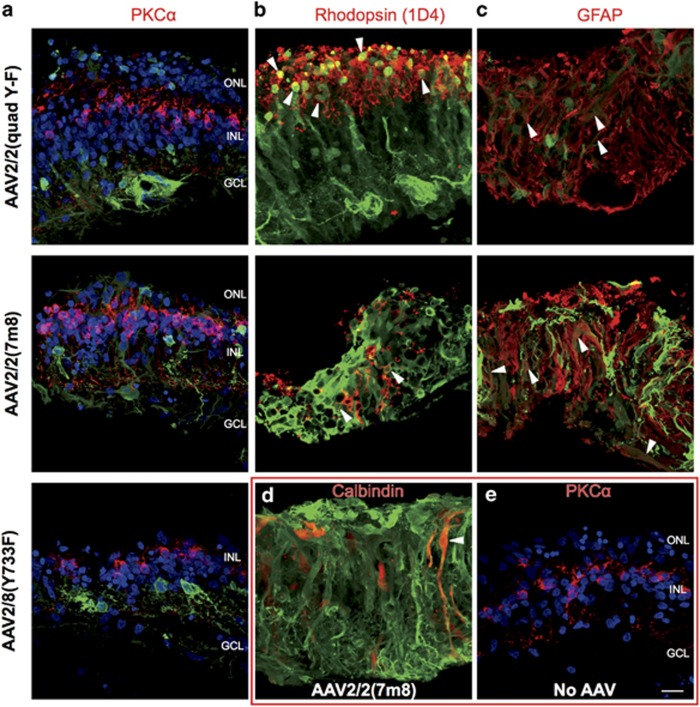
Fluorescence micrographs of human retina explants transduced with either AAV2/8(Y733F)-, AAV2/2(quad Y-F)- or AAV2/2(7m8)-GFP, or no AAV and co-labelled for GFP and (**a** and **e**) protein kinase-Cα (PKCα), (**b**) rhodopsin, (**c**) glial fibrillary acidic protein (GFAP) or (**d**) calbindin. (**a**, **e**) No cells were clearly double labelled for GFP and PKCα. (**b**) Explants transduced with both AAV2/2(quad Y-F)-GFP and AAV2/2(7m8)-GFP were identified as co-labelled for GFP and rhodopsin, suggesting transduced rods (arrowheads). (**c**) AAV2/2(quad Y-F)-GFP and AAV2/2(7m8)-GFP transduced retinas showed GFP colocalisation with GFAP (arrowheads). (**d**) Sections of AAV2/2(7m8)-GFP transduced retina were noted to have cells that were both GFP and calbindin positive (arrowhead). (**e**) No GFP was identified in retina not exposed to either AAV. All sections are oriented with the photoreceptors up. GCL, ganglion cell layer; INL, inner nuclear layer; ONL, outer nuclear layer. Scale bar, 20 μm.

Immunolabelling of transduced human retina samples showed GFP expression across the width of each of the samples transduced with an AAV-GFP vector (Figure 7). Whereas the human retinal explant transduced with AAV2/8(Y733F) showed GFP expression limited to cells largely in the inner nuclear layer (Figure 7a), explants transduced with AAV2/2(quad Y-F) and AAV2/2(7m8) showed extensive GFP expression in all layers of the retina (Figures 7b and c). Rod bipolar cells were identified by immunolabelling PKCα (Figure 8a). No GFP-positive cells were found to be PKCα positive, suggesting very few rod bipolar cells were transduced in these human retinal explants. Rod photoreceptors were transduced, as shown by co-labelling with an anti-1D4 antibody that binds to rhodopsin (Figure 8b). Transduced cones were not identified (red/green opsin and cone arrestin staining; data not shown), but this may be because of the low number of cones in the peripheral retina where these samples were taken from and/or compromised tissue health.

The extensive GFP expression in human retinal explants transduced with AAV2/2(quad Y-F) and AAV2/2(7m8) had an unusual pattern that was not stereotypical of a particular cell type in a normal retina. However, some of the areas of GFP expression that spanned the full thickness of the retina colocalised with glial fibrillary acidic protein (and calbindin), suggesting some of these cells were Müller cells (Figures 8c and d). A control human retinal explant that was not transduced with an AAV-GFP vector did not show any signal in the GFP channel (Figure 8e).

## Discussion

Engineered AAV serotypes have the potential to expand the potency and cell targets of naturally occurring serotypes, thereby facilitating the targeted delivery of gene therapies to previously untreatable cell types and a reduction of therapeutic dose. This study aimed to characterise three potent AAV serotypes as tools for basic and translational research by comparing the cell types transduced and the levels of transduction achieved by intravitreal and subretinal injection of the degenerate mouse eye and transduction of macaque and human retinal explants. Across the three species tested, the AAV2/2(7m8) serotype was the most efficient at transducing a wide area of a given retina and a diverse range of cell types. In the degenerate mouse retina, AAV2/2(7m8) injected either intravitreally or subretinally led to retinas having a large area expressing GFP. AAV2/2(quad Y-F) also transduced consistently across the three species, but not to the extent of AAV2/2(7m8). AAV2/8(Y733F) transduction was more variable and generally the lowest of the three.

The general pattern of GFP expression varied greatly between mouse, macaque and human retinas. This may be because of species-specific tropism factors as well as a combination of the health status of the tissue and/or the experimental procedure. In this regard, it is notable that the macaque tissue was isolated from healthy fresh retina, whereas the human retinal samples were obtained from patients undergoing retinectomy for long-standing retinal detachments. As retinal injury is known to activate Müller cells by expression of glial fibrillary acidic protein,^28^ this may also influence viral tropism. The challenges of intraocular surgery—including variability in the site of injection, the success of retinal detachment and the speed of vector delivery—would have contributed to the variability in the mouse *in vivo* results. This variability mirrors the challenge of delivering AAV to patients.

In the degenerate mouse retina, horizontal cells, retinal ganglion cells and retinal pigment epithelium were the cell types that had the greatest levels of GFP expression, regardless of the AAV serotype injected (Table 1). Watanabe *et al.*^29^ performed subretinal injections of AAV2/2 and AAV2/8 (no capsid mutations) into nondegenerate retinas and also reported that horizontal and ganglion cells were efficiently transduced, whereas amacrine cells were less commonly transduced and bipolar cells were rarely transduced. A previous study by our group demonstrated similar patterns of retinal cell tropism following intravitreal and subretinal injection of AAV2/8(Y733F).^17^

The predominant cell type transduced in the macaque retinal explants was the cone photoreceptor (Table 1). GFP expression was high in cones across the retina in samples that received AAV2/2(7m8)-GFP, whereas GFP expression was greatest at the outer edges of foveal explants that received AAV2/8(Y733F)-GFP or AAV2/2(quad Y-F)-GFP. The lower density of cones in peripheral retinal explants is thought to explain the overall lower level of GFP expression in these samples. Previous studies have reported that wild-type AAV2,^30^ AAV2/2(quad Y-F) and AAV2/2(7m8) intravitreally injected into macaque eyes leads to a ring of fluorescence, centred on the fovea^15^—suggesting a common mechanism leading to poor transduction of the central fovea. The thickness of the inner limiting membrane (ILM) may be a significant factor in limiting foveal transduction *in vivo* and in explant cultures. The ILM forms the interface between the retina and the vitreous and consists of the plasma membrane of Müller cells, a dense basal membrane and loose collagen fibrils extending into the vitreous cortex.^31^ The ILM is much thicker (~400 nm) at the fovea compared with the equatorial region of the retina and surrounding the optic disc, where it is ~70 nm thick,^32^ creating a greater physical barrier to AAV penetration. The ILM role in limiting AAV retinal transduction is supported by data demonstrating that degrading the ILM using proteases^33^ or injection to the sub-ILM space^34^ leads to enhanced retinal transduction.

GFP expression was also seen in the ganglion cell layer, but this was often at the extreme periphery of the macaque explants. It was unclear as to whether this peripheral transduction was facilitated by the absence of the ILM as a barrier to diffusion, or whether the trauma of creating the explant’s cut edge may have enhanced transduction. It is possible that the periphery of each explant effectively mimics the degenerate retina that has a more permeable ILM^26^ or the thinner ILM of a rodent.^33^

The results of this study compare well with those reported by Dalkara *et al.*^15^ In their study, AAV2/2(quad Y-F)-GFP (referred to as AAV2-4YF-CMV-GFP by the authors) and AAV2/2(7m8)-GFP (referred to as 7m8-CMV-GFP by the authors) were injected intravitreally into one eye each of a *Macaca fascicularis* monkey. By 3 weeks after the injection, GFP expression was evident at the fovea, with the AAV2/2(7m8)-GFP eye showing higher levels of GFP expression. Although the current study used *ex vivo* explants rather than *in vivo* injections into a different species (*M. mullata*), and used a CAG promoter rather than the cytomegalovirus promoter used by Dalkara *et al.*,^15^ it is interesting to note the consistent finding of greater GFP transduction by AAV2/2(7m8) compared with AAV2/2(quad Y-F).

Ramachandran *et al.*^27^ injected AAV2/2(7m8) (referred to as AAV7m8 by the authors) by the intravitreal and subretinal routes into the eyes of *M. fascicularis*. At the lowest dose (1 × 10^10^ vg) they observed rods and few cones to be transduced, whereas at higher doses (1 × 10^11^ and 1 × 10^12^ vg) cones were efficiently transduced. These high-dose results are similar to the *ex vivo* retinal explant results of the current study, despite a lower dose (only 10^10^ vg per explant) of AAV. This result may be explained by the fact that AAV is contained in a fixed volume in an explant culture, whereas *in vivo* AAV is effectively diluted by diffusion out of the subretinal bleb and removal via the vasculature. Furthermore, the retinal pigment epithelium removes AAV particles *in vivo*.

The qualitative results obtained from transduction of human retina samples shows that all three AAV serotypes transduced human retinal cells relatively efficiently and that these serotypes are likely to prove effective in targeting retinal neurons *in vivo* (Table 1). Given that these retina samples typically came from patients who had chronic retinal detachments, the transduced areas of retina are likely to have abnormal physiology, and this may explain the unusual patterns of GFP expression in some of the samples. Activated Müller cells, for example, appeared to be strongly transduced by AAV2/2(7m8) and AAV2/2(quad Y-F). Müller cell activation is known to be a feature of proliferative vitreoretinopathy seen in chronic retinal detachment.^35^ Consistent with this explanation, such a pattern of GFP expression was not identified in degenerate mouse or nondegenerate macaque transduced retinal samples.

Consistent with previous studies, it was found that bipolar cells had very low rates of transduction.^36^ Given that retinal bipolar cells are an ideal target for optogenetic gene therapy,^19^ these low rates of bipolar cell transduction are significant. It is likely that the concentration of AAV required to effectively transduce retinal bipolar and amacrine cells is relatively high. Effective bipolar cell transduction using these AAV serotypes may therefore require higher AAV concentrations than tested in this set of experiments. Macé *et al.*^18^ achieved widespread bipolar cell transduction by intravitreally injecting a high-concentration GFP-expressing AAV2/2(7m8) vector into *rd1* mice. The number of vector particles injected by Macé *et al.*^18^ was ∼60 × greater (1.8 × 10^11^ vector particles injected/eye; vg concentration unknown) than the number of viral genomes delivered in the current study (3 × 10^9^ vg/eye). This highlights the requirement for highly concentrated AAV to achieve effective bipolar cell transduction. The use of a bipolar cell-specific—rather than ubiquitous—promoter may also be critical to increasing bipolar cell transduction efficiency, as has been demonstrated in mice.^37^

Transduction of primate bipolar cells has proved more challenging than transducing mouse bipolar cells. Subretinal injection of a high concentration (1 × 10^12^ vg) of AAV8BP2, a capsid customised to bipolar cell transduction in mice, did not efficiently transduce bipolar cells in nondegenerate retina of *M. fascicularis*.^27^ In this study by Ramachandran *et al.*,^27^ minimal bipolar cell transduction was achieved in *M. fascicularis* retina following injection of high doses of AAV2/2(7m8) (1 × 10^11^ and 1 × 10^12^ vg), the higher of which resulted in severe, chronic inflammation.

The discrepancies between the cellular transduction patterns between mice and macaques led Ramachandran *et al.*^27^ to suggest that retinal explants from non-human primates or post-mortem human samples may be more suitable for directed evolution experiments to isolate serotypes suited for human clinical applications. Given the similarity in appearance of the current study’s explant sections and those from *in vivo* injections reported by Ramachandran *et al.*,^27^ this study supports the use of non-human primate explants as a model for directed evolution of primate-optimised AAV serotypes. This approach has the advantages of removing surgical variability and enabling multiple variables to be tested in parallel using tissue from a single animal.

A direct comparison of the intravitreal and subretinal routes of delivery was completed as part of the studies in *rd1* mice. AAV2/2(7m8) and AAV2/2(quad Y-F) both transduced a similar area of the retina regardless of their route of delivery. An important consideration when deciding on a delivery route is the differences in anatomy between the mouse and human eye. The human eye has a vitreous cavity of much greater volume relative to the retinal area, compared with that of a mouse eye. Therefore, the potential for dilution throughout the vitreous cavity following an intravitreal injection is much greater in the human eye. Intravitreal injections additionally expose virions to cellular and extracellular off-target receptors that may capture virions (effectively diluting the dose) and/or initiate adverse effects by binding to elements involved in immune responses. In contrast, a subretinal injection controls the volume through which virions are distributed and limits the range of receptors that the virions are exposed to. Other studies that have injected the same AAV serotype intravitreally into mouse and primate eyes have observed a much lower rate of transduction in the primate eye.^15^

A limitation of this study was that all of the data were gathered at a single time point—3 weeks after injection for the mouse studies and ∼1 week for macaque and human explant studies. Different serotypes can have different rates of expression because of the different ways in which AAV capsids interact with cell surface receptors and the rate at which the vector genome is uncoated.^38^ AAV tropism outcomes have been assessed at 3 weeks post injection in other studies,^17^ whereas others have assessed outcomes in nondegenerate mice at 4 weeks.^10^ Natkunarajah *et al.*^12^ found that i*n vivo* fluorescence from AAV2/8 (no capsid mutations) did not reach a plateau until 7 weeks post injection. Longer-term data would be helpful to contrast with this data set and to ensure that expression is sustained long enough to justify clinical applications.

A further limitation of this study was that only one concentration of AAV vector was investigated. Studies have shown that the number of vector genomes injected can have an important influence on the transduction efficiency and pattern.^14, 39^ The number of vector genomes injected was limited by the lowest concentration of the AAV preparations that were produced. Variability in the size of human retinal explants also meant that the multiplicity of infection could not be standardised. The use of uniform sized discs of macaque retinas minimised multiplicity of infection variability in macaque explants.

In summary, we report that an AAV variant developed by a process of directed evolution, AAV2/2(7m8), effectively transduces a range of cell types when delivered to the degenerate mouse retina by intravitreal or subretinal injection as well as macaque and human retinal explants. The rationally engineered serotypes AAV2/8(Y733F) and AAV2/2(quad Y-F) also transduce multiple retinal cell types across the three species, but to a lesser extent. Differences in the pattern of transduction between mouse and macaque suggest the thickness of the ILM may present a significant barrier to effective retinal transduction following intravitreal injection. These data demonstrate that AAV2/2(7m8) should be considered a valuable vector for developing effective clinical gene therapy strategies.

## Materials and methods

### Plasmids

A plasmid containing humanised GFP (GenBank accession number U50963.1) driven by a ubiquitous CAG (*C*MV enhancer, chicken β-*a*ctin, rabbit β-*g*lobin) promoter was a gift from Bill Hauswirth, University of Florida (Gainesville, FL, USA).^40^

Plasmids containing the *rep*-*cap* genes to make AAV2/8(Y733F) and AAV2/2(quad Y-F) AAV serotypes were created by site-directed mutagenesis, based on published mutation sites.^9, 41^ The *rep*-*cap* plasmid to produce AAV2/2(7m8) was a gift from John Flannery, University of California, Berkeley.^15^ The 3.23 kb single-stranded sequence was packaged into three different AAV capsids: AAV2/8(Y733F), AAV2/2(quad Y-F) and AAV2/2(7m8).

### AAV production and titration

Recombinant AAV was produced using a triple transfection of 293T cells using polyethylenimine followed by density separation by an iodixanol gradient and buffer exchange using phosphate-buffered saline (PBS). Vector genome concentration was determined by quantitative PCR comparison with plasmid standards following DNase I (New England Biolabs, Ipswich, MA, USA) digest and heat denaturation of AAV. For further details see Supplementary Methods.

### Ethical review

Animal experiments were performed in accordance with the United Kingdom’s Animals (Scientific Procedures) Act 1986. Experiments were performed at a Home Office-approved site (code: 30/2306) and under the purview of a project licence (30/2808) that was approved and periodically reviewed by the University of Oxford’s Clinical Medicine Animal Welfare and Ethical Review Body and the Home Office.

The acquisition of human retina tissue from patients with retinal detachment was approved by the Berkshire Research Ethics Committee, part of the National Health Service’s National Research Ethics Service (REC Code 10/H0505/8).

### Animals

C3H/HeNCrl female mice (referred to as ‘*rd1* mice’) were purchased from Charles River (Wilmington, MA, USA). Mice were housed in individually ventilated cages under a 12 h light (<100 lux)/dark cycle with the cage temperature set to 21±3 °C and 55±10% relative humidity. Pellets of RM3 diet (Special Diets Services, Witham, UK) and water were available *ad libitum*.

### General anaesthesia

Mice were anaesthetised using 80 μg g^−1^ of body mass of ketamine (Vetalar, Zoetis, Florham Park, NJ, USA) and 10 μg g^−1^ xylazine (Rompun, Bayer AG, Leverkusen, Germany) administered via intraperitoneal injection using a 1 ml insulin syringe and 29 G needle (Terumo, Tokyo, Japan). Ketamine and xylazine were mixed together with the appropriate volume of sterile water.

Anaesthestic reversal was achieved by intraperitoneal injection of 2 μg g^−1^ of body mass of atipamezole (Antisedan, Orion Corporation, Espoo, Finland), made up with sterile water.

### Intraocular injections

Under general anaesthesia, each eye of female 13–14-week-old C3H/HeNCrl mice was injected with ∼3 × 10^9^ vg of one of three different serotypes of AAV-GFP. One eye received an intravitreal injection, the other a subretinal injection. Leakage of AAV from one eye to the contralateral eye has not been demonstrated,^42, 43^ and hence each eye was treat as independent. Four eyes were injected for each combination of AAV serotype and delivery route. In the absence of data quantifying the effect size, the sample size was chosen based on the sample size of comparable studies.^14, 17^ Mice were selected randomly for injection with a given serotype. To minimise the risk of contamination, mice were injected with the same serotype. The surgeon was masked to the AAV serotype injected. Further analysis was done in an unmasked manner.

After the induction of general anaesthesia, 1% (w/v) tropicamide and 2.5% (w/v) phenylephrine (Bausch & Lomb, Bridgewater, NJ, USA) were applied to dilate the pupils. Carbomer gel (Viscotears; Alcon, Hünenberg, Switzerland) was applied to both eyes and a 6∼mm diameter cover glass (VWR International, Radnor, PA, USA) placed on the gel to enable visualisation of the fundus.

AAV solution was thawed on ice and diluted to the appropriate concentration with PBS, when necessary. Using a 5 μl syringe (65 RN, Hamilton Company, Reno, NV, USA) and a 34 G, 10 mm long, point style 2 needle (Hamilton Company), 1–1.5 μl of AAV solution was aspirated with a 0.5 μl air bubble between the solution and the distal tip of the plunger. The air bubble aided visualisation of the injection site.

An anterior chamber paracentesis was performed using a 1 ml insulin syringe with a 29 G needle before subretinal injections to reduce ocular pressure to aid globe manipulation and intraocular injection. Subretinal vector delivery was performed under direct visualisation using an operating microscope (Leica Microsystems, Wetzlar, Germany). The needle was advanced through the sclera, choroid and retinal pigment epithelium into the subretinal space. The injection site was posterior to the equator and typically superotemporal in the left eye and superonasal in the right eye. When the needle tip was visualised as being subretinal, the AAV solution was injected. The appearance of air bubbles under the retina confirmed a subretinal injection. Any mixed subretinal/intravitreal injections were excluded. The area of each retina exposed to a subretinal injection was documented by manual drawings.

The procedure for an intravitreal injection did not include anterior chamber paracentesis. The needle was passed through the sclera, choroid and retina and into the vitreous cavity, where the solution was injected. Seeing an air bubble against the posterior surface of the lens confirmed the injection was intravitreal. The needle was left *in situ* for at least 30 s to allow the intraocular pressure to normalise.

All injection wounds were allowed to self-seal. Following intraocular injection, drops of 0.5% (w/v) chloramphenicol and 0.5% (w/v) proxymetacaine hydrochloride (Bausch & Lomb) were applied to both eyes. Mice were placed in a chamber warmed to ∼35 °C for the duration of their recovery from anaesthesia. Mice were monitored for signs of ocular or systemic health issues in the hours and days after an injection.

Between injections with the same AAV solution the needle and syringe were flushed at least 10 times with sterile saline solution. Between different AAV solutions the needles and syringe were flushed at least 20 times.

### Confocal scanning laser ophthalmoscopy

Mice were imaged using a Spectralis HRA (Heidelberg Engineering, Heidelberg, Germany) with general anaesthesia.

Pupillary dilation was achieved by the application of a 1:1 mixture of 1% (w/v) tropicamide and 2.5% (w/v) phenylephrine (Bausch & Lomb) at least 5 min before imaging. A drop of hypromellose BPC 0.3% (w/v) (Martindale Pharma, Romford, UK) followed by a custom-made contact lens was applied to each cornea to prevent dehydration and cataract formation.^44^

In near-infrared mode (820 nm laser) with a 55° lens, the focal plane was adjusted to the layer of maximum reflectance and an image acquired. Changing to autofluorescence mode (488 nm excitation laser, 500–700 nm emission detection), a series of images were acquired at sensitivity settings 40, 50, 60, 70, 80 and 90. The automated real-time feature, without normalisation, was used to improve image quality. In near-infrared mode the focal plane was moved to the inner retina to acquire images of the ganglion cell layer in near-infrared and autofluorescence modes. Images of other areas of interest were acquired, but not using standardised settings.

### Quantification of scanning laser ophthalmoscopy images

Images were exported from Eye Explorer software (Heidelberg Engineering) as 1536 × 1536 pixel TIFF images. Images acquired at a sensitivity setting of 70 were compared. Using ImageJ^45^ the images were converted to 8-bit. A circular region of interest outlining the whole 55° field of view was overlaid on each image. A minimum pixel value threshold was set to 30. This value was chosen as it included all areas that were subjectively judged to be transduced. The minimum threshold value was kept constant across all images. The measure function was used to calculate the percentage of the area within the region of interest that was above the threshold and the mean pixel value of the area above the threshold. These data were copied into Excel (Microsoft, Redmond, WA, USA) for analysis. One eye with media opacity was not included in the quantitative analysis.

### Tissue collection

Mice were killed by cervical dislocation. Immediately following the confirmation of death the eyes were enucleated. The cornea was punctured using a 29 G needle (Terumo) and the eye immersed in 4% formaldehyde in PBS at 4 °C overnight. After 16–24 h, the eye was rinsed in PBS for ∼1 min before being immersed in 30% sucrose for at least the time required for it to become negatively buoyant.

### Embedding and sectioning of eyes and explants

Tissue that had been formaldehyde fixed and immersed in 30% sucrose was briefly washed in PBS and transferred to a mould containing Optimum Cutting Temperature (OCT; Tissue Tek, Sakura Finetek, Tokyo, Japan). After 5 min, the tissue was placed on pellets of dry ice. Once frozen the sample was stored at −80 °C.

Samples were sectioned using a cryostat (LM1850, Leica Biosystems, Wetzlar, Germany). Cryostat temperature was typically between −20 °C and −25 °C. Sections were 20 μm thick and were applied to Polysine slides (Menzel-Gläser, Braunschweig, Germany).

Sections were left to dry at room temperature under metal foil for at least 1 h before being stored at −20 °C.

### Immunohistochemistry: sections

Slides that had been stored at −20 °C were dried at room temperature for at least 1 h. Tissue was permeabilised with 0.2% Triton X-100 (Sigma-Aldrich, St Louis, MO, USA) in PBS for at least 20 min at room temperature. Sections were rinsed with PBS for at least 1 min before blocking with 10% donkey serum in 0.2% Triton X-100 in PBS for at least 1 h at room temperature. Primary antibodies were diluted (Table 2) in 2.5% donkey serum and 0.2% Triton X-100 in PBS. Slides were placed in a humidified box and placed at 4 °C overnight. When double or triple labelling the two or three, respectively, primary antibodies were mixed together and applied in a single solution.

Sections were washed by covering in 0.05% Tween-20 in PBS for at least 10 min. This was repeated four times. Secondary antibodies were diluted 1:200 in 2.5% donkey serum and 0.2% Triton X-100 in PBS. Slides covered with secondary antibody solution were placed in a humidified dark box and incubated at room temperature for 2 h.

Sections again underwent four washes with 0.05% Tween-20 in PBS for at least 10 min for each wash. One final wash was completed with water. Slides were mounted with ProLong Gold with DAPI mountant (Invitrogen, Waltham, MA, USA) and placed in a dark box at room temperature overnight. After the mountant had set, the slides were stored at 4 °C.

### Immunohistochemistry: flat mounts

Visualised through a dissecting microscope the retina was removed from the eyecup and transferred to 30% sucrose in a microcentrifuge tube.

To facilitate antibody penetration, the retina was freeze-fractured using liquid nitrogen. The sample was placed in liquid nitrogen and left to freeze. The tube was removed and thawed at room temperature. Degenerate retinas underwent one freeze–thaw cycle, whereas nondegenerate retinas had two cycles.

Retinas were permeabilised by immersion in 1% Triton X-100 for 10 min, repeated three times. Retinas were blocked by immersion in 10% donkey serum for at least 2 h (typically overnight). Primary antibodies were diluted in 2.5% donkey serum and 1% Triton X-100 in PBS. Retinas were incubated in primary antibody solution for up to 3 days at 4 °C.

Retinas were washed with 0.2% Triton X-100 in PBS three times, with each step lasting at least 30 min on an orbital shaker. Secondary antibodies were diluted 1 in 200 with 2.5% donkey serum and 1% Triton X-100 in PBS and incubated in the dark overnight at 4 °C.

Retinas were washed with 0.2% Triton X-100 in PBS four times, each step lasting at least 40 min on an orbital shaker. During the fourth wash step Hoechst 33342, diluted 1 in 5000 in PBS, was added. A final wash was done with water. Visualised under a dissecting microscope the retina was cleaned of debris and cuts were made to enable it to be flattened. The retina was transferred to a Polysine slide, with the ganglion cell layer facing up. The retina was dried before applying ProLong Gold mountant. A coverslip was lowered onto the retina and bubbles displaced. The slide was left in the dark at room temperature overnight for the mountant to set before being stored in the dark at 4 °C.

### Image acquisition

Micrograph images were acquired using an inverted epifluorescence (Leica Microsystems) or an inverted confocal (Carl Zeiss, Oberkochen, Germany) microscope. Settings were optimised to minimise signal interference and avoid image saturation. Where quantitative or semiquantitative comparisons between samples were to be made, the acquisition settings were kept constant.

Using ImageJ, minimum and maximum pixel values of the whole image were adjusted to aid visualisation of relevant features in figures.

### Confocal imaging

Slides that had undergone the slide immunohistochemistry protocol in parallel were imaged on an inverted confocal microscope under standardised conditions. Using a 40 × oil immersion lens, two sections from each slide were imaged. Two slides per eye were imaged, providing representative coverage across the eye. Images were acquired from areas of high fluorescence that were not at the extreme periphery of the retina, adjacent to optic nerve or at an obvious injection site. Within the section the imaging plane was set to that what gave the greatest total fluorescence, as judged subjectively. The pinhole was set to 1 Airy unit for the green channel. For image snaps that were to be compared, laser power, gain, speed, averaging and image resolution were kept constant after being set to minimise pixel saturation.

Using ImageJ, each image was manually segmented into the inner nuclear layer, inner plexiform layer and ganglion cell layer using the polygon selection tool. A threshold minimum pixel value was set and the measure tool used to calculate the percentage area and mean pixel value of each of the three regions of interest of each image, as previously described.^17^ This objective technique is less susceptible to subjective cell counting errors and has comparable outcomes.^46^ These data were copied into Excel. The mean of the percentage area and pixel value from the multiple images of a given eye (technical replicates) was used as the value to compare between eyes of different animals (biological replicates).

### Macaque retinal explants

Rhesus macaque (*M. mullata*) eyes were obtained from the MRC Centre for Macaques (Porton Down, UK). Tissue was collected post mortem from male and female macaques ranging in age from 5 to 19 years (mean: 14.8 years old) undergoing planned killing for other purposes. After sedation with 10 mg kg^−1^ intramuscular ketamine and a lethal overdose of 200–300 mg kg^−1^ intravenous pentobarbitone, the eyes were immediately enucleated. In a dish of complete neurobasal A (see below) the cornea and lens were removed and radial incisions made to flatten the retina. A 3 mm diameter biopsy tool was used to take discs of neuroretina from the fovea and from around the vascular arcades (referred to as peripheral samples). Retinal pigment epithelium separated from the neuroretina and hence was not transferred. Using a transfer pipette (Sarstedt, Nümbrecht, Germany) each retinal disc explant was transferred to a tube containing chilled complete neurobasal A that was stored in a cooled polystyrene box for transport to the laboratory (a period of 2–6 h).

In the laboratory, each retinal disc was placed in a tissue culture insert (0.4 μm pore size, VWR International) in a well of a 24-well plate. Then, 500 μl of complete neurobasal A was placed in each well below the insert and 200 μl in the insert. Explants were cultured at 34 °C, 5% CO_2_.

Complete neurobasal A was made of penicillin (final concentration: 100 units per ml), streptomycin (100 μg ml^−1^; Sigma-Aldrich), L-glutamine (800 μM; Sigma-Aldrich), B-27 supplement (2% Gibco, Waltham, MA, USA) and N-2 supplement (1% Gibco) in neurobasal A (Gibco).

Two biological replicates were used for each AAV serotype and for both foveal and peripheral samples.

### Human retinal explants

Human retina samples were obtained from consented patients in an ethics approved clinical study, in which retinal tissue was being removed as part of their standard care. The surgery involved a limited retinectomy for retinal detachment at the Oxford Eye Hospital. Patients were aged 39, 60 and 83 years. Depending on the surgical requirements the retinectomy was performed by the vitreoretinal surgeons with ophthalmic surgical scissors or with a 20 G or 23 G vitrector. Samples were removed from the eye using a backflush flute needle and ejected into a sterile sample bottle containing balanced salt solution.

Within 1 h, samples were placed into a tissue culture insert (0.4 μm pore size) placed in a well of a 24-well plate. Then, 400 μl of complete neurobasal A was placed in each well below the insert and 300 μl placed in the insert. Explants were cultured at 34 °C, 5% CO_2_.

Two biological replicates were used for each AAV serotype. Technical replicates were not possible because of the limited amount of tissue available.

### AAV transduction of primate retinal explants

Approximately 24 h after retina explant cultures began, the media were replaced with complete neurobasal A and a volume of AAV containing 10^10^ vg to a total volume of 600 μl (final concentration 1.67 × 10^10^ vg per ml). Explants were incubated at 34 °C, 5% CO_2_. Media were replaced every 2 days. The porosity of the membrane on which the explants rested meant that it was assumed that explants were surrounded by AAV on all surfaces. Investigators were not masked to which AAV serotype was applied.

### Fixation of retinal explants

At 7 days after AAV was applied, explants were fixed. Explants were washed in PBS and fixed in 4% formaldehyde (Thermo Fisher Scientific, Waltham, MA, USA) in PBS for 2 h at room temperature. Following another wash in PBS, explants were placed in 30% sucrose (w/v in PBS; Sigma-Aldrich) and stored at 4 °C.

### Statistical analysis and graphing

Statistical analysis and chart preparation was completed using Prism (GraphPad, San Diego, CA, USA).

When comparing two independent variables, with at least two conditions and a single dependent variable, an ordinary two-way ANOVA was used. The *post hoc* tests were conducted with Bonferroni’s multiple comparisons tests. When available, sample groups were all compared with a single control group. When not available, all groups were compared with each other. The significance level was α=0.05 for all tests.

Graphical data are presented as mean±s.e.m.

## Figures and Tables

**Table 1:** Semiquantitative summary of transduction patterns of three AAV serotypes delivered intravitreally and subretinally to degenerate mouse retinas and to macaque and human retinal explants

	*Retinal pigment epithelium*	*Horizontal cells (calbindin)*	*Rod bipolar cells (PKCα)*	*Amacrine cells (calbindin, GAD67, GlyT1)*	*Retinal ganglion cells (Brn3a)*	*Photosensitive retinal ganglion cells (mOPN4)*
*Degenerate mouse retinas*						
AAV2/8(Y733F) Intravitreal	+	+	+/−	+	+	
AAV2/8(Y733F) Subretinal	++	+++	+/−	+	++	
AAV2/2(quad Y-F) Intravitreal	++	++	+/−	+	++	
AAV2/2(quad Y-F) Subretinal	++	+++	+/−	+	++	
AAV2/2(7m8) Intravitreal	++	++	+/−	+	+++	+
AAV2/2(7m8) Subretinal	++	+++	+/−	+	++	

	*Rods (rhodopsin)*	*Cones (calbindin)*	*Rod bipolar cells (PKCα)*	*Amacrine cells (calbindin, GlyT1)*	*Retinal ganglion cells (Brn3a)*	
*Nondegenerate macaque retinal explants*						
AAV2/8(Y733F)	+/−	+	+/−	+	+	
AAV2/2(quad Y-F)	+/−	++	+/−	+	+	
AAV2/2(7m8)	+/−	+++	+/−	+	++	

	*Rods (rhodopsin)*	*Cones (calbindin)*	*Rod bipolar cells (PKCα)*	*Müller cells (GFAP)*		
*Human retinal explants*						
AAV2/8(Y733F)	−	−	−			
AAV2/2(quad Y-F)	+	−	−	++		
AAV2/2(7m8)	+	−	−	++		

Abbreviations: AAV, adeno-associated virus; GAD67, glutamate decarboxylase 67; GFAP, glial fibrillary acidic protein; GlyT1, glycine transporter 1; PKCα, protein kinase-C-α.

The cell marker antibody used to identify the cell type is indicated in parentheses. +/−, occasional transduction; +, consistent, weak transduction; ++, strong, consistent transduction; +++, very strong transduction.

**Table 2:** Details of primary antibodies

*Staining*	*Immunogen*	*Host species*	*Source (product code)*	*Clonality*	*Dilution*
Brn3a	Epitope mapping near the N terminus of Brn3a of human origin	Goat	Santa Cruz (sc-31984)	Polyclonal	1:1000
Calbindin	Full-length native protein corresponding 28 kDa calbindin-D protein purified from rat kidney	Rabbit	Abcam (ab11426)	Polyclonal	1:5000
GAD67	[1G10.2]. Recombinant GAD67 protein	Mouse	Millipore (MAB5406)	Monoclonal	1:500
GFAP	GFAP isolated from cow spinal cord	Rabbit	Abcam (ab7779)	Polyclonal	1:1000
GFP	Recombinant full-length protein	Chicken	(1) Aves (GFP-1010); (2) Abcam (ab13970)	Polyclonal	1:1000
GlyT1	Synthetic peptide from the carboxy-terminus as predicted from the cloned rat GLYT1	Goat	Millipore (AB1770)	Polyclonal	1:1000
OPN4	Amino acids 1–15 mapping to the N terminus of melanopsin of mouse origin (UF006)	Rabbit	Advanced Targeting Systems (AB-N38)	Polyclonal	1:2500
PKCα	[Y124]. Synthetic peptide corresponding to residues in C terminus of human PKCα	Rabbit	Abcam (ab32376)	Monoclonal	1:1000
Red/green opsin	Recombinant human red/green opsin	Rabbit	Millipore (AB5405)	Polyclonal	1:1000
Rhodopsin (1D4)	C-terminal 9 amino acids of mammalian rhodopsin (TETSQVAPA)	Mouse	Gift from Dr Jill Cowing	Monoclonal	1:1000

Abbreviations: GAD67, glutamate decarboxylase 67; GFAP, glial fibrillary acidic protein; GFP, green fluorescent protein; GlyT1, glycine transporter 1; PKCα, protein kinase C-α.
