# Supplementary material for: Tropism of engineered and evolved recombinant AAV serotypes in the rd1 mouse and ex vivo primate retina
Source: Gene Ther. 2017 Nov 16;24(12):787–800. doi: 10.1038/gt.2017.85 (PMC5746594; doi:10.1038/gt.2017.85)
Supplement: Supplementary Methods [file gt201785x4.docx]

**Supplementary Methods**

Recombinant AAV was produced by a triple transfection of 293T cells followed by iodixanol purification, buffer exchange and concentration.

293T cells were grown to approximately 80% confluency in a T175 flask (Greiner Bio-One) before being resuspended, trypsinised, centrifuged and resuspended in DMEM. Approximately 35 million 293T cells were seeded into a hyperflask (Sigma-Aldrich) filled with complete DMEM. Two hyperflasks were seeded for each AAV preparation. Hyperflasks were incubated at 37 °C in a 5% CO_2_ humidified environment for two to three days, until cells were 60 – 80% confluent.

Cells were triple transfected with a total of 500 µg of DNA per hyperflask using polyethylenimine (PEI; Polysciences) transfection agent. The three plasmids mixed in a 1:1:1 molar ratio were a helper plasmid (pAdΔF6) containing essential adenovirus helper genes required for AAV production ^46^, a *rep*-*cap* plasmid containing the AAV replication and capsid encoding genes, and a CAG-GFP expression cassette plasmid with AAV2 inverted terminal repeats.

The three plasmids were mixed in a 50 ml conical centrifuge tube and the volume made up to 10 ml with 150 mM sodium chloride (NaCl). In a separate 50 ml tube, 1.125 ml of 1 mg/ml PEI (pH 7.0) was made up to 10 ml with 150 mM NaCl. The PEI mixture was added to the plasmid mixture. The complete mixture was incubated at room temperature for approximately 20 minutes.

Transfection mixture was poured into a 500 ml bottle of DMEM made up with 2% fetal bovine serum, 2 mM L-glutamine, 100 units/ml penicillin and 100 µg/ml streptomycin. This media was used to replace the hyperflask media. The hyperflask was returned to a 37 °C, 5% CO_2_ incubator for three days.

When the transfected cells were confluent, approximately 200 ml of media was poured out of the hyperflask. The hyperflask was vigorously shaken to detach the cells. The hyperflask was emptied into a sterile 500 ml bottle (Nalgene PPCO centrifuge bottle, Thermo Scientific) and centrifuged at 180 g for 10 minutes at 20 °C (Heraeus Megafuge 16R, Thermo Scientific). Most of the supernatant was poured off, leaving a poorly defined cell pellet and approximately 30 ml of media, which was transferred to a 50 ml conical centrifuge tube. Centrifuging this at 180 g for 10 minutes at 20 °C produced a well-defined cell pellet. The media was poured off and the pellet resuspended in 15 ml of lysis buffer and 500 µl of protease inhibitor solution (one protease inhibitor cocktail tablet (F. Hoffman-La Roche) dissolved in 1 ml of lysis buffer). The lysed cell pellet was stored at -80 °C.

Lysed cell pellet was thawed in a 37 °C water bath for 15 minutes and returned to -80 °C for at least an hour. This freeze-thaw cycle was repeated three times. After the final thaw, benzonase nuclease (Merck Millipore) was added to a final concentration of 50 U/ml and incubated in a 37 °C water bath for 45 minutes, mixing every 15 minutes. Centrifugation at 3,700 g for 20 minutes at 20 °C enabled separation of AAV-containing supernatant from the pellet of cell debris.

Iodixanol gradients were made in ultracentrifuge tubes (Optiseal, Beckman Coulter) by layering 15% (7.2 ml), 25% (4.8 ml), 40% (4 ml) and lastly 60% (4 ml) iodixanol fractions below one another into an ultracentrifuge tube using a UV-sterilised 3.5 ml transfer pipette (Sarstedt). Using a transfer pipette the AAV-containing supernatant was transferred to the top of an iodixanol gradient, filling the ultracentrifuge tube entirely. Three iodixanol gradients were required for each AAV preparation. Full ultracentrifuge tubes were transferred into an ultracentrifuge rotor (Type 70Ti, Beckman Coulter) and spun at 358,300 g for 90 minutes at 20 °C (Optima XE-90 Ultracentrifuge, Beckman Coulter). Acceleration and deceleration were set to maximum.

Using an 18 G needle (BD Microlance 3, Becton, Dickinson and Company) attached to a 10 ml syringe (BD Plastipak, Becton, Dickinson and Company) the ultracentrifuge tube was punctured at the junction of the 40% and 60% fractions and the AAV-containing 40% fraction was slowly aspirated and ejected into a 50 ml conical centrifuge tube and stored at 4 °C overnight.

In the final stage, iodixanol was replaced with a saline buffer and AAV was concentrated by reducing the final volume. AAV loss was minimised by use of a centrifuge tube containing a removable cellulose filter that binds proteins with a molecular weight greater than 100 kDa (Amicon Ultra-15, Merck Millipore). First, the filter was washed by adding 5 ml of saline (PBS or BSS with 0.001% Pluronic F-68 (PF68; Gibco)) and centrifuging at 3,000 g for 15 minutes at 20 °C. The AAV-containing iodixanol was thoroughly mixed with 5 ml of saline and transferred to the washed filter. Centrifuging at 3,000 g at 20 °C continued until the volume was reduced to approximately 1 ml. A further 15 ml of saline was mixed with the residual volume and centrifuged at 3,000 g at 20 °C until the volume was again reduced to approximately 1 ml. The addition of 15 ml of saline was repeated twice more. For the final centrifuge step the volume was reduced to 250 – 500 µl to concentrate AAV particles in a small volume. Using a P200 pipette the filter was washed by passing the remaining AAV solution over the filter at least 20 times. Concentrated AAV was aliquoted in 10 and 20 µl volumes into 500 µl nuclease-free tubes. When necessary, AAV solutions were diluted using the same saline used for purification.

AAV titre was determined by quantitative PCR and capsid proteins were visualised by SDS-PAGE.
